# Dislocation–Twin Boundary Interactions Induced Nanocrystalline via SPD Processing in Bulk Metals

**DOI:** 10.1038/srep08981

**Published:** 2015-03-11

**Authors:** Fucheng Zhang, Xiaoyong Feng, Zhinan Yang, Jie Kang, Tiansheng Wang

**Affiliations:** 1 State Key Laboratory of Metastable Materials Science and Technology, Yanshan University, Qinhuangdao 066004, China

## Abstract

This report investigated dislocation–twin boundary (TB) interactions that cause the TB to disappear and turn into a high-angle grain boundary (GB). The evolution of the microstructural characteristics of Hadfield steel was shown as a function of severe plastic deformation processing time. Sessile Frank partial dislocations and/or sessile unit dislocations were formed on the TB through possible dislocation reactions. These reactions induced atomic steps on the TB and led to the accumulation of gliding dislocations at the TB, which resulted in the transition from coherent TB to incoherent GB. The factors that affect these interactions were described, and a physical model was established to explain in detail the feasible dislocation reactions at the TB.

Many studies explored the nanocrystallization mechanisms during the severe plastic deformation (SPD) of various metals, including pure Fe^1^,^2^, Cu and Cu alloys^3^,^4^,^5^, stainless steels^6^,^7^, Ni and Ni alloys^8^,^9^,^10^,^11^, and Al and Al alloys^12^. These mechanisms principally include dislocation activities (dislocation nucleation, slipping, and reaction), twinning, and their interactions. For simplicity, the mechanism strongly depends on the stacking fault energy (SFE) and the crystal structure of metals^13^,^14^,^15^,^16^. Grain refinement in metals with high SFE is dominated by dislocation activities under low strain rate conditions^5^,^8^,^17^. For metals with low SFE and under the condition of low strain rate or low temperature in small grain sizes, grain refinement mainly proceeds with the formation of deformation twins and subsequent twin–twin intersections^13^,^18^. Moreover, dislocation–twin boundary (TB) interactions reportedly play important roles in grain refinement^8^. These interactions may be a type of grain refinement mechanism for metals with medium SFE.

Deformation twinning usually occurs simultaneously with the slip of unit and partial dislocations; TBs also serve as sites for dislocation nucleation and accumulation^19^,^20^,^21^,^22^. Thus, gliding dislocation–TB interactions inevitably occur in grains. These interactions are significantly affected by dislocation density^23^, twin thickness, Schmid factor on different slip planes or the orientation of a crystalline grain^24^,^25^, energy barrier for dislocation reactions^23^,^26^, and SFE^27^,^28^, among others. For example, high SFE complicates the nucleation and the slip of partial dislocations, which weaken the interactions between individual partials and TBs. The energy barrier is calculated by subtracting the total energy of the initial dislocations from the energy of the dislocation produced by the reaction and determines the feasibility of the reaction. Furthermore, face-centered cubic metals have been found to deform via twinning more readily than their coarse-grained counterparts^29^,^30^,^31^, this increases the probability of interactions between dislocations and twins. These reports raise certain questions: Is the dislocation–TB interaction a type of grain refinement mechanism in most metals? If it is, what are the possible dislocation reactions at TB? How then do the extrinsic factors affect the interactions during nanocrystallization?

To answer these questions, Hadfield steel (X120Mn12) with a medium SFE^32^, i.e., 48 mJ/m^2^ 33, was subjected to high speed pounding (HSP)^34^,^35^. The microstructural evolution of the steel during nanocrystallization was characterized by transmission electron microscopy (TEM) and high–resolution transmission electron microscopy (HRTEM). Results show that the dislocation–TB interactions are an essential grain refinement mechanism. The interactions and the factors that affect these interactions are described in detail.

## Results

**Figure 1:**
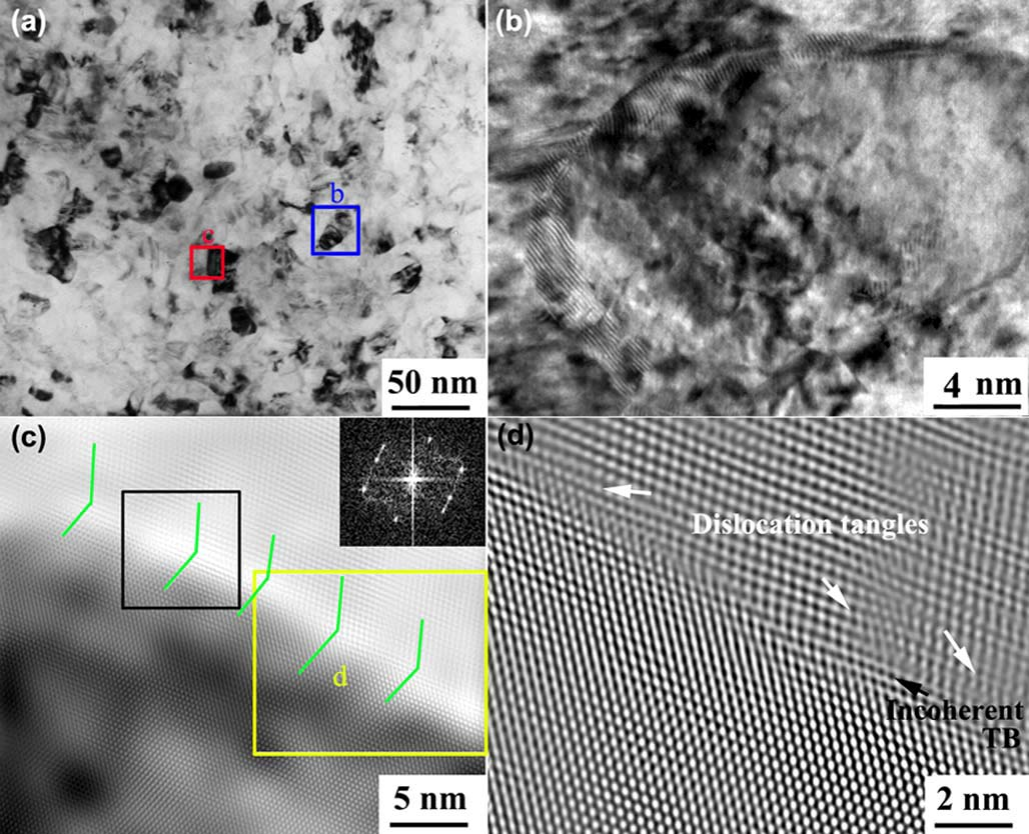
Nanocrystalline boundary structure of sample surface subjected to HSP for 8 × 10^4^ times: (a) equiaxed nanocrystalline; (b) a close view of area “b” in Fig. 1(a); (c) a close view of area “c” in Fig. 1(a) and corresponding fast Fourier-transformed image in a black rectangular box; (d) inverse fast Fourier-transformed image of area “d” in Fig. 1(c).

A nanocrystalline surface layer formed after HSP was performed 8 × 10^4^ times (Fig. 1). A close view of area “b” in Fig. 1(a) reveals the presence of several dislocations and the absence of twinning, as shown in Fig. 1(b). HRTEM observation (Fig. 1c) on the boundary between adjacent nanograins was performed to clarify dislocation–TB interactions. The green lines indicate the location of (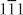) planes on both sides of a (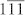) TB. Two grains in the nanoscale share a twin relationship, as indicated by the fast Fourier-transformed image in Fig. 1(c). The results reveal that the feasible dislocation–TB interactions cause the TB to disappear and turn into a grain boundary (GB) during nanocrystallization. Fig. 1(d) shows the inverse fast Fourier-transformed image of area “d” in Fig. 1(c). As shown in the area, the presence of the curved TB is associated with some unit dislocations. Meanwhile, multiple dislocation tangles or stacking faults occur around the TB, leading to the transition from coherent TB to incoherent high-angle GB.

**Figure 2:**
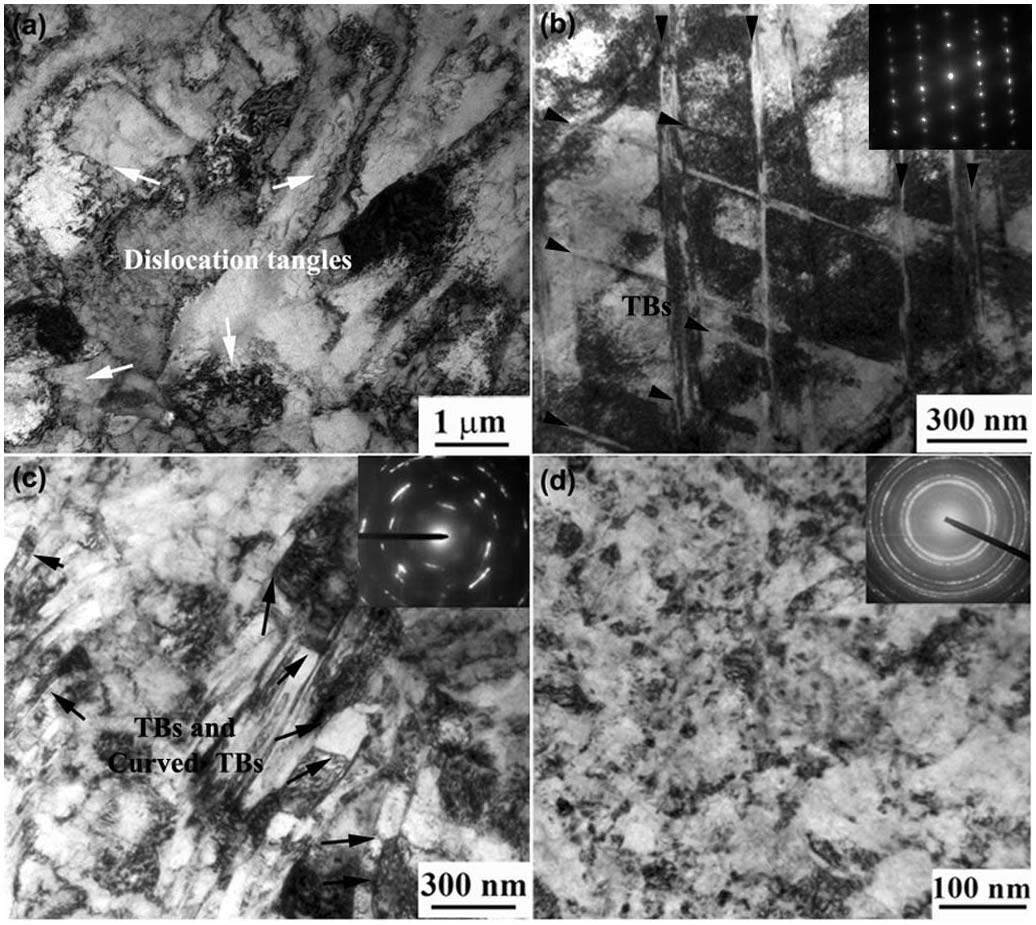
TEM images of samples subjected to HSP for different times: (a) 1 × 10^4^, (b) 2 × 10^4^, (c) 4 × 10^4^, and (d) 8 × 10^4^ times.

To characterize the microstructural evolution during HSP, a series of TEM observations on the samples subjected to HSP for different times is shown in Fig. 2. At the early stage of SPD, dislocation activities are the dominant deformation mechanism. High density dislocations in the Hadfield steel are arranged in tangles and groups (Fig. 2a). As pounding times increase, twins and intersections of twins start to populate the microstructure resulting in rhombic blocks, as indicated by selected area electron diffraction pattern and marked by black arrow (see Fig. 2b). The twinning then becomes the dominant deformation mechanism, even though the dislocation activities are also active (Fig. 2b). Subsequently, high-density deformation twins break up the original grains, and the dislocations are trapped between the TBs. Therefore, these dislocations are impaired from gliding freely, and forced interactions with the TB are inevitable. Fig. 2(c) presents an arrangement of nanoscale twins that form cells and twins that grow inside other twins. The presence of TBs and curved TBs has been frequently observed, as marked by black arrow. High density dislocation glide and either accumulate at TBs or transmit across TBs lead to the formation of curved TBs. The curved TBs indicate that the disorientation between the twin and the matrix is gradually increases and is not completely random yet, i.e. these cells have similar but not equal orientations. The dislocation–TB interactions can trigger the formation of equiaxed nanograins with high-angle GBs in the final stage of nanocrystallization (Fig. 2d).

**Figure 3:**
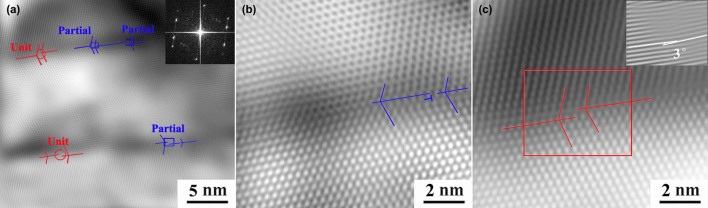
HRTEM images showing dislocations at the TB of the sample subjected to HSP for 4 × 10^4^ times: (a) macroscopic view of twins; (b) a close view of Frank partial dislocation at the TB; (c) a close view of unit dislocation at TB and corresponding 1D Fourier-filtered image of the red-boxed selected area. Continuous lines indicate the location of the axis of symmetry of twins. Red circles show the location of steps in the TB associated with unit dislocation, whereas blue rectangular boxes indicate the location of Frank partial dislocation.

Figures 1 and 2 reveal that the nanocrystallization process includes the disappearing of deformation twins, which is found to be the ubiquitous in the SPD process. The dislocation–TB interactions are the leading grain refinement mechanism in Hadfield steel. Considering that this finding answers the aforementioned first question, we attempted to find the answers to the other questions. A close observation of the dislocations at the TB is shown in Fig. 3. A narrow twin (≈20 nm) is clearly observed in Fig. 3(a). Detailed analysis of the image reveals the presence of numerous dislocations, which introduce steps at the TB. Fig. 3(b) shows the presence of Frank partial dislocation at the TB with a Burgers vector of 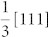. A unit dislocation is present at the TB with a Burgers vector of 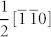, thereby inducing a step with a height of one (111) atomic layer (Fig. 3c). Importantly, the glide plane of the unit dislocation is not parallel to any {111} glide plane in both the twin and the matrix. This result indicates that the unit dislocation is sessile. A 1D Fourier-filtered image of the selected area marked in Fig. 3(c) shows a disorientation angle of approximately 3° between the left and right parts of the step at the TB, which is marked in Fig. 3(c) by two white lines parallel to the (111) of the twin and the matrix. These sessile dislocations at the TB cause distortion at the core of the dislocations, thereby breaking the coherence of the atomic arrangement at the TB.

## Discussion

**Figure 4:**
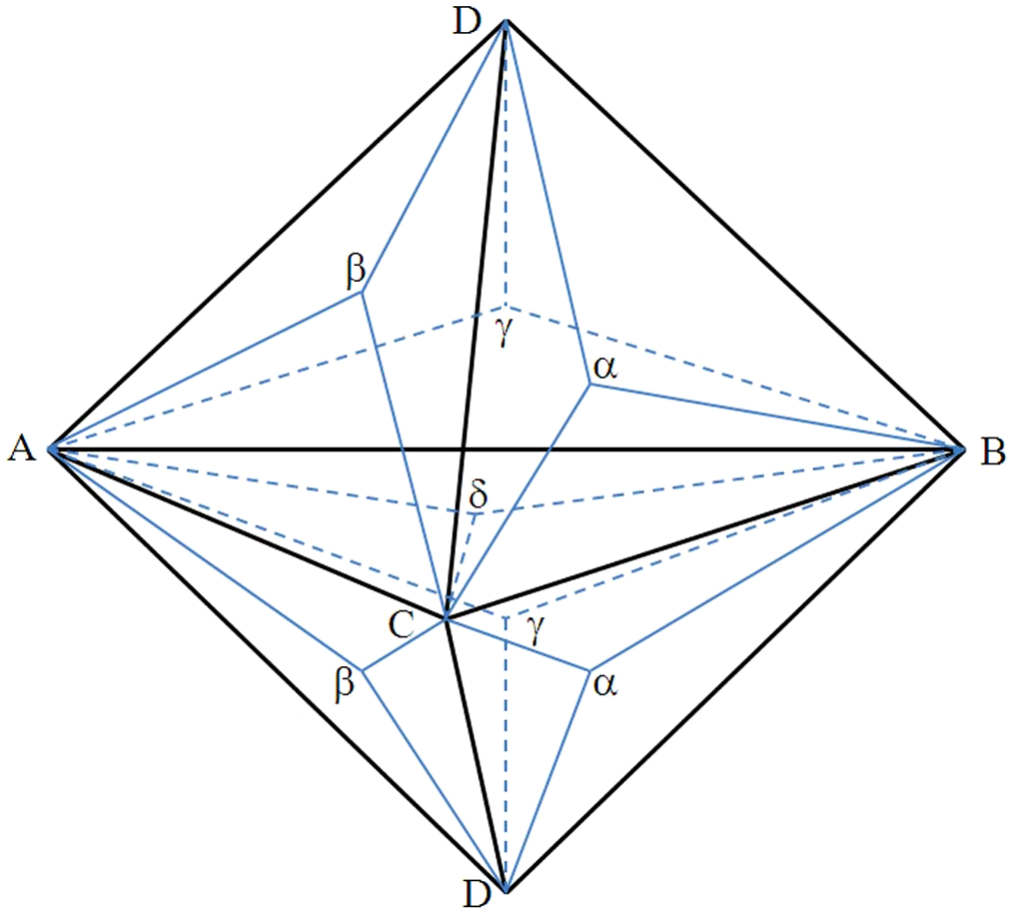
Illustration of a double Thompson tetrahedron.

To conveniently discuss the feasible dislocation–TB interactions, we introduce a double Thompson tetrahedron in Fig. 4. The TB is also a (111) slip plane, i.e., the ABC plane in the Thompson tetrahedron. The Thompson tetrahedron above the (111) TB represents the slip system in the matrix, whereas the bottom tetrahedron represents the slip system in the twin. Given that the other three slip planes are identical close-packed planes to gliding dislocations, we assume that the dislocation glides on the ACD plane toward the TB for ease of discussion. The dislocation reactions that produce Frank partial dislocation (Dδ) are described as follows: (1) a unit dislocation AD reacts with a partial δA on the ABC plane, and a Frank sessile dislocation δD is formed; (2) the unit dislocation AD glides on the ACD plane toward the ABC plane, dissociating into two partials, Aδ and δD. The reactions are as follows:
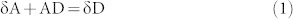

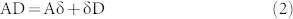


The presence of a sessile Frank partial dislocation hinders glissile Shockley partials from slipping along or inclined to TB. The former can react with a Frank partial to produce a unit dislocation and induce a sessile incoherent TB step. The reaction can be described as follows:
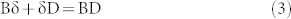


The feasibility of these reactions is discussed by calculating the energy barrier for dislocation reactions at the TB as described in the literature^23^,^26^. The energy barrier for reactions (1), (2), and (3) are −2.5 *Ê* 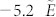, 2.4 *Ê*, and −2.4 *Ê*, respectively, where 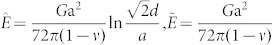, in which *G* is the shear modulus, *ν* is Poisson's ratio, *d* is the grain size, and *a* is the lattice parameter. *Ê* and 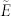 are positive values. The occurrence of the reaction depends on whether or not the energy barrier can be overcome. The energy barriers for reactions (1) and (3) show negative values, which mean that they are energetically favorable and can occur spontaneously during SPD. Obviously, the two reactions reduce the dislocation density.

**Figure 5:**
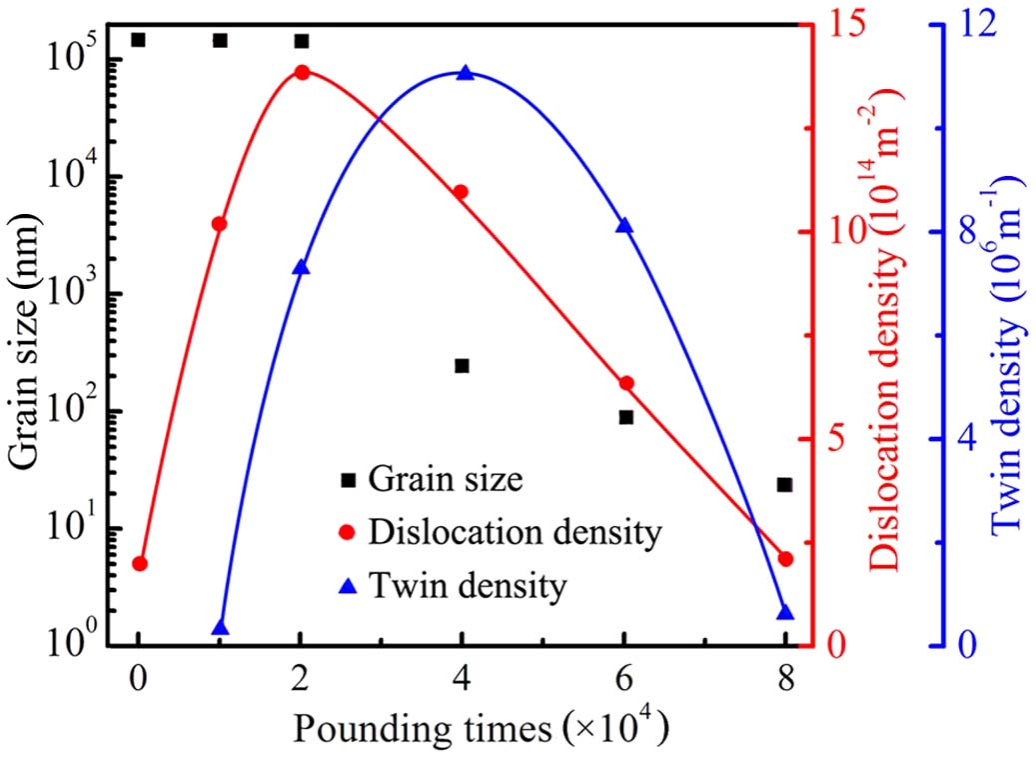
Grain size, dislo, and twin density as functions of HSP processing time. Grain size measured from the TEM images of a large amount of samples; dislocation density estimated using X-ray diffraction analysis with the Williamson–Hall method^39^; twin density calculated from the overall length of the TB from a large number of samples divided by the overall TEM image area.

Aside from energy barrier, twin thickness and dislocation density are also expected to affect the dislocation–TB interactions. Fig. 5 shows that the grain size, dislocation density, and twin density vary with the SPD processing time. At the early stage of SPD, the dislocation and twin densities increase. The TB acts not only as the barrier to dislocation motion but also as the site for dislocation accumulation^19^,^36^,^37^. Thus, high dislocation and twin densities can enhance the flow stress and increase the driving force that activates dislocation–TB reactions. With the increase in twin density and dislocation–TB interactions, dislocation reactions increase, dislocation annihilation increases, and dislocation density decreases. The low thickness (nanoscale) of the twinning lamella is accompanied by increased strain and twin density. When the twin thickness decreases to approximately 200 nm, more dislocation–TB interactions contribute to the observed grain refinement because the refined twinning lamella reduces the critical stress required by the nucleation and slipping of Shockley partial dislocations at the twinning steps or the junctions of TB and GB^38^. Consequently, the twin density decreases. The dislocation–TB interactions cause the TB to disappear and turn into a high-angle GB, which is the dominating nanocrystallization process in Hadfield steel.

**Figure 6:**
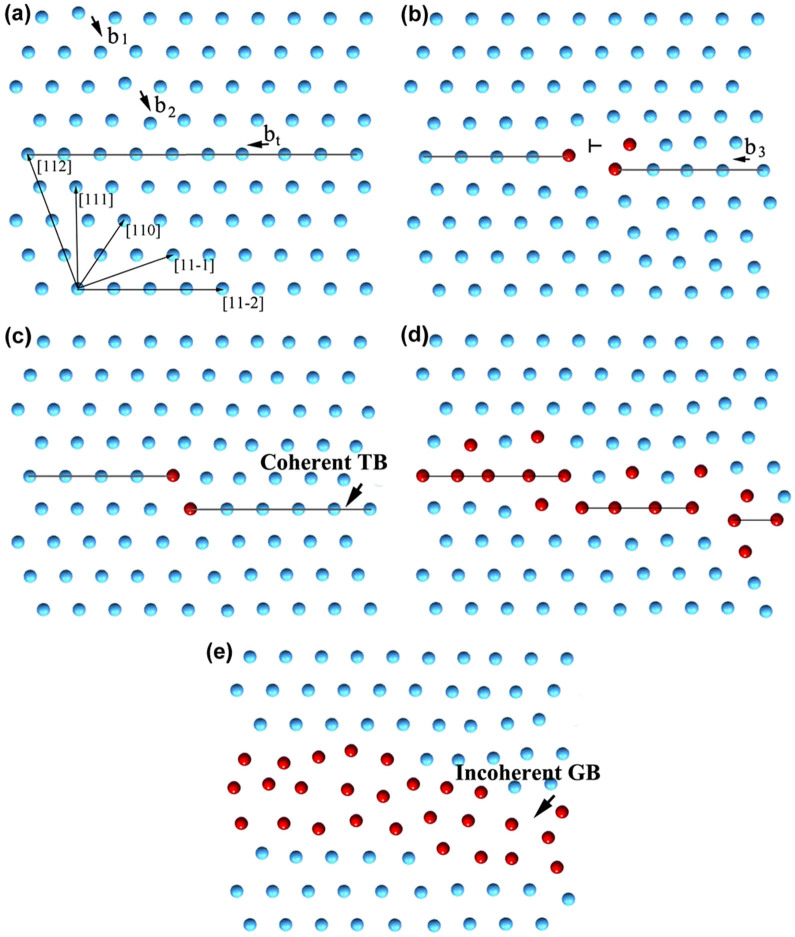
Schematic of nanocrystallization resulting from dislocation–TB interactions.

For simplicity, we propose a physics-based model of the nanocrystallization mechanism for Hadfield steel subjected to SPD resulting from the dislocation–TB interactions (Fig. 6). An extended dislocation slipping on the adjacent {111} plane of the TB, which consists of two glissile Shockley partials (with Burgers vectors *b_1_* and *b_2_*) and a short stacking fault between the two partials, constricts and forms a unit dislocation when its motion is blocked by the TB (Fig. 6a). Frank sessile dislocation occurs when the unit dislocation reacts with a glissile Shockley partial slipping (Burgers vector, *b_t_*). Meanwhile, a step and incoherent atomic arrangement in the core of sessile dislocation are formed at the TB (Fig. 6b). A glissile Shockley partial dislocation (*b_3_*) that approaches the step along the TB reacts with the sessile dislocation to form a sessile unit dislocation while forming a step with one (111) atomic layer height (Fig. 6c). Notably, the presence of incoherent atomic arrangement at the TB is associated with dislocation accumulation.

As SPD continues, more sessile unit dislocations are formed at the TB, thereby forming steps and leading to the TB morphology transition from flat to rough. Meanwhile, these sessile unit dislocations can act as barriers to the dislocation motion on the adjacent {111} plane, causing dislocation reaction and tangling. In addition, the steps at the TB are assumed to act as stress concentration sites that trigger dislocation emission, as suggested by molecular dynamic simulations and experimental observations^40^,^41^. This action exacerbates the accumulation of the gliding dislocations in the twins. Therefore, the coherence of the TB is gradually broken, and a local stress concentration is triggered in the area; these phenomena activate more dislocation reactions and lead to the formation of a disorientation angle at the TB (Fig. 6d). When the dislocations–TB interactions repeatedly occur at the TB, the morphology is altered from flat to curved. As a result, a coherent TB eventually becomes a curved and incoherent GB, as shown in Fig. 6(e).

The dislocation activities and twinning are the dominant deformation mechanisms in the SPD of Hadfield steel. However, the dislocations do not form dislocation cells that break up the grains into subgrains, as frequently observed in certain high-SFE metals, such as Fe^1^,^2^, Ni^9^,^11^ and Al^12^. Twin–twin intersections occur but without concomitant martensite transformation, as observed in certain low-SFE materials, such as AISI stainless steel^7^. These observations suggest that the dislocation–TB interactions are the dominant nanocrystallization mechanism in Hadfield steel.

In summary, our study demonstrated that the dislocations–TB interactions result in the disappearing of TB and turning to high angle GB in the SPD–processed Hadfield steel. Large plastic deformation generates more sessile unit dislocations at the TB and induces atomic steps, leading to an accumulation of the gliding dislocations at the TB and the transition from coherent TB to incoherent GB. The findings in this report complement the nanocrystallization process originated from the deformation twins, which may provide a guide help on preparing nanocrystalline in some metals, especially in medium SFE metals whose the dominant deformation mechanism is twinning and dislocation activities.

## Methods

### Sample preparation and SPD technology

The materials used in this investigation were Hadfield steel plates (25 mm thick) with chemical compositions of (in wt.%): 1.20 C, 12.30 Mn, 0.60 Si, 0.016 S, 0.022 P, and balance Fe. Prior to SPD, the samples were treated at 1050°C for 60 min and quenched in water to obtain a uniform austenitic microstructure with grain size ranging between 100 and 200 μm. HSP was carried out as the SPD technology. The HSP set-up and procedures were described in our previous papers^34^,^35^. During HSP process, the surface layer of sample was plastic deformation with gradient distributions of applied strain rate and strain. In the top surface, the estimated strain rate was about 2 × 10^2^ s^−1^. In the present work, the samples were subjected to HSP at room temperature for 1 × 10^4^, 2 × 10^4^, 4 × 10^4^, and 8 × 10^4^ times respectively.

### Characterization techniques

The microstructures of processed samples were examined using TEM and HRTEM (JEM-2010, operating at 200 kV). The samples for TEM were prepared by slicing a thin foil parallel to the plane of the pounded surface, followed by polishing the sample from the non-pounded side into ~30 μm and subsequently thinning to perforation using a TenuPol–5 twin–jet instrument. The X-ray diffraction (XRD, D/max-2500/PC) profile was recorded. The Williamson–Hall method^39^ was used to estimate the dislocation density. We also define the twin density as the TB length in unit area. Thus, we calculated the overall length of the TBs from more than 20 TEM images with observation field of several μms, and then divided the length by the overall TEM image area.

## Author Contributions

F.C.Z. and X.Y.F. proposed the idea and designed the research plan. X.Y.F. carried out the experiments and collected the data. F.C.Z., X.Y.F. and Z.N.Y. analysis the data and wrote the paper. J.K. and T.S.W. performed TEM investigation.
